# Energy-dispersive X-ray spectroscopy and atom-probe tomography data quantifying component-ratios of multicomponent nano-precipitates in ion-irradiated ceria

**DOI:** 10.1016/j.dib.2021.107460

**Published:** 2021-10-08

**Authors:** Karen Kruska, Weilin Jiang, Xuemei Wang, Lin Shao, Brian J. Riley, Ram Devanathan

**Affiliations:** a Energy and Environment Directorate, Pacific Northwest National Laboratory, P.O. Box 999, Richland, WA, 99352, USA; b Department of Nuclear Engineering, Texas A&M University, 3133 TAMU, College Station, TX, 77843-3133, USA

**Keywords:** Multicomponent alloy, ε-phase, STEM EDS, Atom-probe tomography, Surrogate fuel, Ion irradiation, Heat treatment

## Abstract

Samples of ∼1 µm films of CeO_2_ doped with 2 wt% Mo, 1.5 wt% Ru, 0.75 wt% Pd, 0.5 wt% Re and 0.25 wt% Rh grown with pulsed laser deposition were irradiated with I^2+^ ions (610 °C and 730 °C, 10^16^ and 5 × 10^16^ I^2+^/cm^2^). For selected samples post-irradiation heat treatment was conducted (900 °C, 1100 °C). The specimens were sectioned with focused ion beam milling and characterized in a transmission electron microscope with energy-dispersive x-ray spectroscopy, and with atom-probe tomography. Energy-dispersive x-ray spectroscopy was used to obtain elemental maps showing the distribution of dopants in the specimen after exposure. Some of these maps are discussed in detail in our companion article “Formation of multicomponent alloy particles in doped ceria under I2+ ion irradiation and thermal annealing” in the Journal of Nuclear Materials [1]. Advanced computational analysis could be used to more accurately quantify local compositions. Data is provided for additional regions of interest and one additional irradiation condition.

The doped ceria film that was heat treated at 1100 °C delaminated from the substrate in most places. Samples were extracted from the underside of a delaminated piece and analyzed with atom-probe tomography. The resulting data show ceria and a Mo-rich particle and demonstrate that this approach is feasable in principle to study local compositions in a sample exposed to such extreme conditions.

## Specifications Table

**Table:** 

Subject	Fuel Technology
Specific subject area	Formation of multicomponent alloys that can function as a possible waste form for ^99^Tc
Type of data	Spectrum Image 3D Ion Map
How data were acquired	Transmission electron microscope: JEOL JEM-ARM 200CF with a Centurio energy dispersice x-ray detector Atom-probe: Cameca LEAP 4000HR
Data format	Raw and Analyzed
Parameters for data collection	The TEM was operated at 200 kV, 15 µA in STEM mode. All atom-probe data was collected at a set temperature of 40K in laser mode with a laser pulse energy of 120 pJ and a pulse rate of 125-200 kHz. The target evaporation rate was set between 0.2 and 0.4%.
Description of data collection	Eenergy dispersive X-ray data was collected using the Thermofisher Scientific Pathfinder software. Atom-probe data was collected with Cameca Integrated Visualization and Analysis Software (IVAS).
Data source location	Institution: Pacific Northwest National Laboratory City/Town/Region: Richland, WA Country: USA Latitude and longitude (and GPS coordinates, if possible) for collected samples/data: 46.34, −119.28
Data accessibility	With the article and in: Repository name: Mendeley Data Data identification number: https://doi.org/10.17632/h38dhp558x.2 Direct URL to data: https://data.mendeley.com/datasets/h38dhp558x/2 Instructions for accessing these data: Instructions on how to open these data are in the description of the dataset at: http://dx.doi.org/10.17632/h38dhp558x.2
Related research article	Karen Kruska, Weilin Jiang, Xuemei Wang, Lin Shao, Brian J Riley, Ram Devanathan, Formation of multicomponent alloy particles in doped ceria under I^2+^ ion irradiation and thermal annealing, Journal of Nuclear Materials 545, 152638 (2021) https://doi.org/10.1016/j.jnucmat.2020.152638

## Value of the Data

• These data provide insight into the formation and resulting composition of multicomponent precipitates in a fuel surrogate material under extreme conditions.
• These data provide knowledge about the conditions necessary to facilitate the formations of such multicomponent particles. This information is crucial if such an alloy is to be used to generate a stable waste form for the volatile ^99^Tc.
• These data show the evolution of precipitates and ejection of these precipitates from the matrix at high temperature (1100°) in a future experiment this can be used systematically to harvest precipitates. The atom-probe tomography (APT) data provided shows that analysis of the precipitates at the delaminated interface is feasible in principle. This technique is very well suited to quantify even very low concentration constituents.
• Computational methods for analysis of spectroscopy data are evolving rapidly. Each of the provided datasets contains much more information than can be reasonably extracted in one image or one graph. Principle component analysis can be used to improve the signal to noise ratio in spectrum maps like the ones provided. Machine learning algorithms need large amounts of data to be trained. These raw data can be used for training of such computational models. Advanced analysis techniques may be able to extract additional information that has been missed.

## 1 Data Description

### 1.1 Energy-dispersive X-ray spectroscopy (EDS) data

Scanning transmission electron microscopy (STEM) EDS maps were acquired from the as-deposited doped ceria film as well as six samples exposed to irradiation and/or high temperature. A summary of the exposure conditions is provided in Table 1. All raw data sets are hosted with Medeley data [2].

**Table 1:** Summary of sample irradiation and heat treatment conditions. Some irradiated samples were annealed weeks after the irradiation experiment to reveal the cumulative effect of irradiation and high temperature. Modified from [1].

Sample ID	Irradiation fluence (I^2+^/cm^2^)	Irradiation temperature (°C)	Annealing temperature (°C)	Annealing time (h)
Sample 1	no irradiation	no irradiation	-	-
Sample 2	10^16^	610	-	-
Sample 3	5 × 10^16^	730	-	-
Sample 4	5 × 10^16^	610	-	-
Sample 5	no irradiation	no irradiation	900	10
Sample 6	5 × 10^16^	610	900	10
Sample 7	5 × 10^16^	610	1100	10

The dataset sample 1 contains one EDS map. The magnification was chosen so that potential 5–10 nm precipitates should be visible in the data. A cracked grain boundary was used for focusing.

The dataset sample 2 contains two EDS maps. The magnification for both maps was also chosen so that potential 5–10 nm precipitates should be visible in the data. In each case a cracked grain boundary was used for focusing.

The dataset sample 3 contains two EDS maps. The corresponding STEM high-angle annular dark-field (HAADF) images show bright contrast indicating the presence of metal precipitates and dark contrast indicating cavities. Analysis of local spectra reveals high Mo- and Pd-concentrations in the precipitates.

The dataset sample 4 contains three EDS maps. The corresponding STEM HAADF images show cracked grain boundaries, but no bright contrast. However, analysis of local spectra revealed small Pd-rich precipitates and I segregation.

The dataset sample 5 contains six EDS maps. Regions were chosen because the corresponding STEM HAADF images revealed dark and bright contrast. Analysis of the local spectra revealed the presence of Mo- and Pd-rich precipitates. Two maps show precipitates formed at the top surface of the film.

The dataset Sample 6 contains four EDS maps. Regions were chosen because the corresponding STEM HAADF images revealed dark and bright contrast. Analysis of the local spectra revealed the presence of Mo- and Pd-rich precipitates. One map shows precipitates formed at the top surface of the film.

The dataset Sample 7 contains seven EDS maps. Regions were chosen close to the interface where many precipitates were located. Analysis of the local spectra revealed the presence of two-phase precipitates with a Mo-rich and a Pd-rich phase.

### 1.2 APT Data

**Fig. 1:**
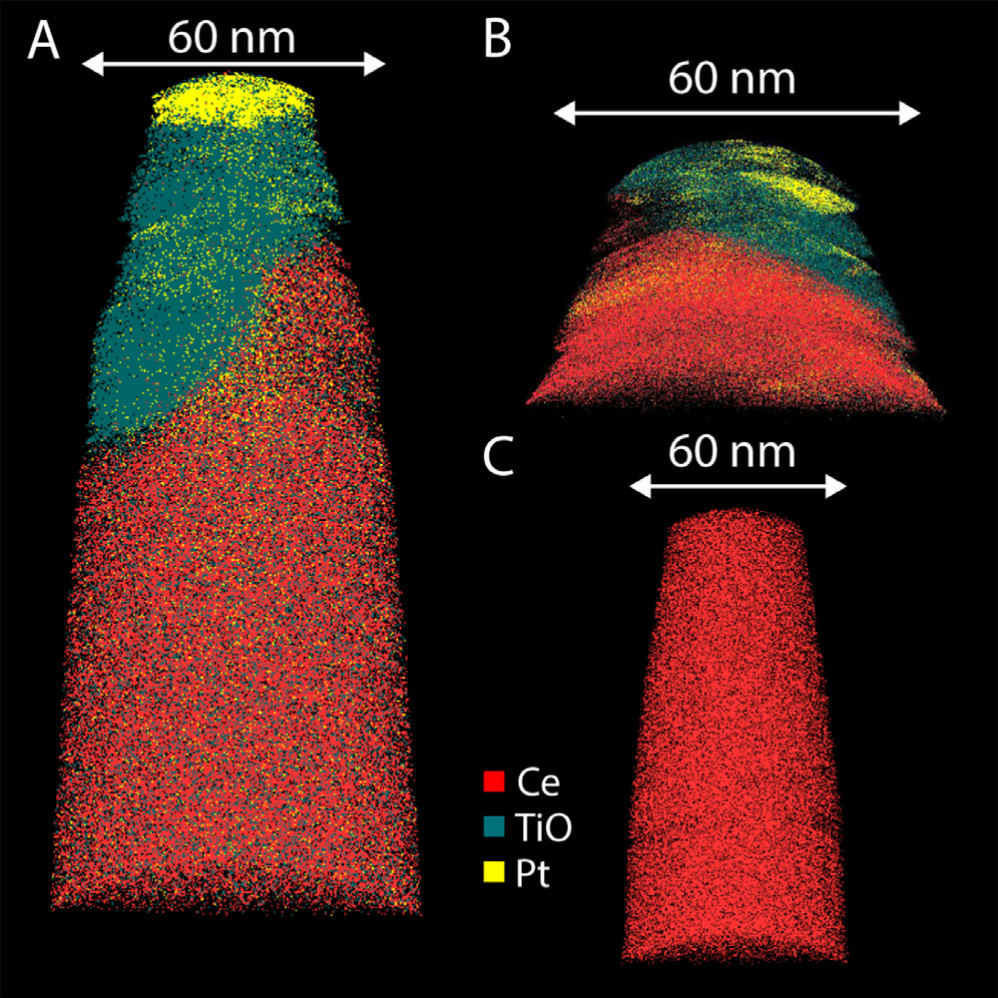
APT ions maps. A) R31_11553. B) R31_11378. C) R31_11556.

Five atom- probe datasets were collected from 4 APT tips. Datasets R31_11378 and R31_11381 were collected from the same tip. All data corresponds to Sample 7. The STEM HAADF images in [1] show that the CeO_2_ film delaminated from the yttria-stabilized zirconia (YSZ) substrate and that there is a high density of precipitates present at the interface. A delaminated flake was mounted upside down and coated with ∼100 nm Ti before sectioning for APT. Although data was acquired and APT has an extremely high chemical sensitivity the mass spectra in the five datasets did not show evidence of the five dopant metals: Mo, Pd, Ru, Re and Rh. Fig. 1 shows ion maps for three of the five datasets. Fig. 1A and B show some of the Ti coating at the surface and the area immediately underneath. Fig. 1C shows a region just below the Ti coating and only contains ceria.

A reconstruction of R31_11378 is shown in Fig. 1B. Near the top the dataset has mostly the Ti coating and some Pt from the focused ion beam (FIB) sample preparation. No precipitates were observed.

The dataset R31_11381 is from the same tip as R31_11378 and contains only Ti coating material.

The dataset R31_11556 contains matrix material at the top; it fractured and a part of the Si micropost was analyzed in the lower half of the dataset

A reconstruction of the dataset R31_11553 is shown in Fig. 1A. The top part contains Ti and Pt and the bottom part contains ceria matrix. The range file used for the reconstruction Ceria_11553.rrng is also included.

A reconstruction of the dataset R31_11556 is shown in Fig. 1C. The dataset only contains ceria matrix.

## 2 Experimental Design, Materials and Methods

To simulate an irradiated UO_2_ fuel, it was necessary to create a non-radioactive material with similar mechanical, high temperature properties and grain structure. Furthermore, this non-radioactive material needed to include fission products (or suitable non-radioactive substitutes) that would form multicomponent alloy particles in UO_2_. CeO_2_ has been established as an appropriate surrogate for UO_2_ [3] because they have the same cubic crystal structure with nearly identical lattice parameters and similar physical and chemical properties. Similarly, Re has been adopted as a substitute for radioactive Tc [4]. The method of choice to create a sample with all the desired elements (95 wt% CeO_2_, 2 wt% Mo, 0.75 wt% Pd, 0.25 wt% Rh, 1.5 wt% Ru, and 0.5 wt% Re) in the correct ratios was pulsed laser deposition (PLD). A deep ultraviolet pulsed KrF exciplex laser (Coherent COMPexPro 102) with a wavelength of 248 nm and a pulse frequency of 5 Hz was used to vaporize the target material and create the plasma plume. The energy density at the target was ∼2.4 J cm^−2^. To force the doped ceria to adopt a polycrystalline grain structure, a polycrystalline YSZ substrate was selected. The lattice mismatch between the two materials is ∼5% and it was found that grain boundaries usually translated from the substrate to the doped ceria film. The YSZ with a purity of 99% was sourced from Marketech International (Port Townsend, WA). For PLD, a target with custom composition matching the desired combination of elements was acquired from Plasmaterials, Inc. (Livermore, CA). The 50.8 mm diameter, 3.175 mm thick target had a purity of 99.9% and all impurities were in the order of tens of ppm or less. The YSZ was annealed in oxygen at 10^–2^ Torr in the deposition chamber to remove any hydrocarbon contamination from the substrate without upsetting its stoichiometry. PLD deposition of oxide materials is often carried out using oxygen as a filtering gas. For this experiment, it was, however, essential to maintain the dopant metals in their metallic form. Initial studies where the PLD was carried out in vacuum showed segregated droplets within the films. To avoid the formation of such droplets in the plume, and ultimately the films, Ar was chosen as an alternative filtering gas. The deposition of the sample film was carried out (after evacuation to 10^–8^ Torr) in Ar at 10^−2^ Torr at 550 °C resulting in 0.2 nm of growth of a somewhat O deficient doped ceria film per second up to a total thickness of ∼1 µm.

**Fig. 2:**
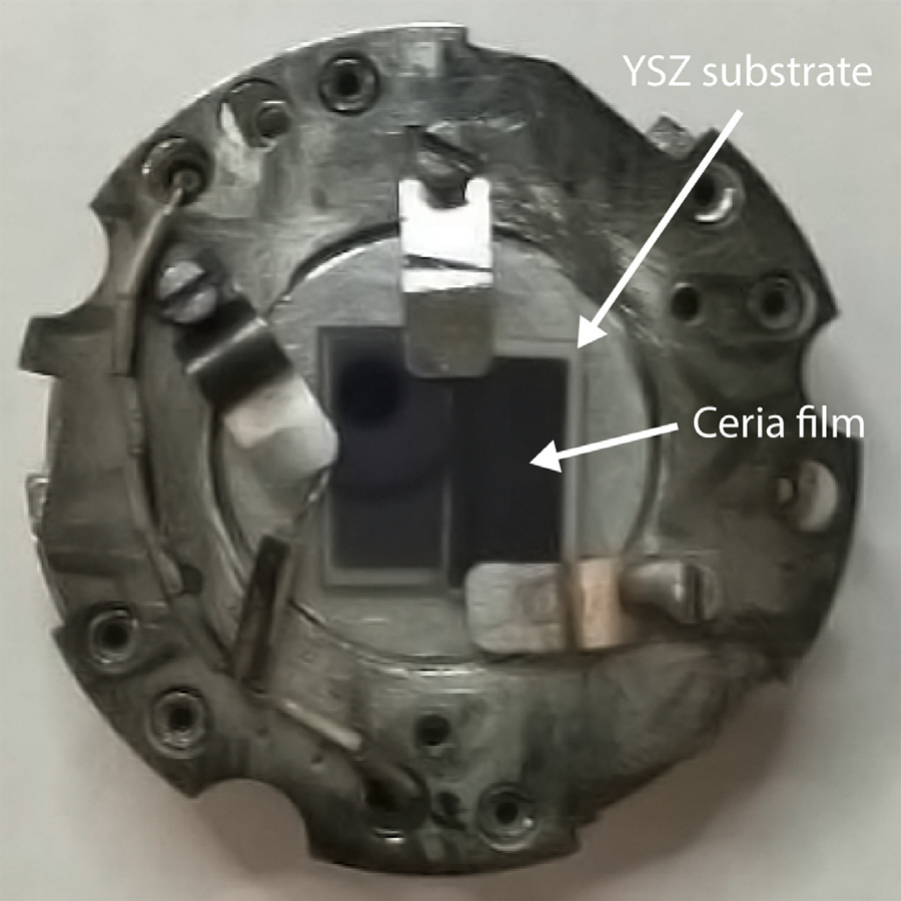
Doped Ceria films on YSZ substrate mounted for ion implantation.

This study utilized ion irradiation to simulate the damaging effect of neutron irradiation without activating the surrogate material. Radiation damage causes the displacement of atoms and hence the creation of defects, such as dislocation lines, loops, and cavities. These defects influence the mechanical, thermodynamic, and kinetic properties of the material. Ion irradiation causes similar damage to neutron irradiation, albeit in a more localized region. Neutron bombardment of nuclear fuel also causes the fission reaction and generation of energy (heat), leaving behind fission products. In this study, a selection of metallic fission products was included in the film as dopants. ^127^I – the stable iodine isotope – was used for I^2+^ ion irradiation to simulate the presence of ^129^I, which is present in irradiated nuclear fuel due to fission of ^235^U. Ion irradiation was performed at Texas A&M University using 2 MeV I^2+^ ions to fluences of 10^16^ and 5 × 10^16^ I^2+^/cm^2^ at 610 °C and 5 × 10^16^ I^2+^/cm^2^ at 730 °C. These temperatures are at the low end of what is relevant to oxide fuels in light water reactors [5]. Fig. 2 shows how two samples were mounted for I^2+^ ion irradiation at 610 °C to 5 × 10^16^ I^2+^/cm^2^. The Stopping and Range of Ions in Matter simulation for I^2+^ in CeO_2_ with a bulk density ρ = 7.215 g/cm^3^ [6] can be found in the accompanying research paper [1]. In the simulation, the threshold displacement energies of E_d_(Ce) = 56 eV and E_d_(O) = 27 eV were adopted [7]. For 2 keV I^2+^ ion implantation at normal incidence, the peak disordering rate is 41.4 atomic displacements per atom (dpa) per 10^16^ I^2+^/cm^2^ at a depth of 294 nm, and the I profile is peaked at a depth of 443 nm with a maximum concentration of 0.39 at.% I per 10^16^ I^2+^/cm^2^. This maximum is close to the center of the ∼1 µm thick film.

A summary of the experimental conditions can be found in Table 1. Some of the samples (Samples 2-4) were characterized in their as-irradiated condition. Unirradiated Sample 5 and Samples 6 and 7 were subjected to an additional heat treatment at 900 °C and 1100 °C. These temperatures correspond to the temperatures expected towards the center of a nuclear fuel pellet in a light water reactor. The heat treatment had to be carried out in a clean environment without O to avoid oxidation of the dopant metals. To this effect, specimens were placed in a cylindrical glassy carbon crucible, which, in turn, was placed in a fused quartz ampoule. The glass ampoules were purged with Ar three times, evacuated to ∼10^−2^ Torr, and sealed with an oxypropane torch. The heat treatments were performed in a box furnace with a temperature ramp of 2 °C/min during heating and cooling, and a 10 h dwell time at the maximum temperature. After heat treatment, the glass ampoules were broken to extract the samples in the crucibles for characterization. While removing the samples from the glass ampoules, it became apparent that the doped ceria films were delaminating from the substrates after the 1100 °C heat treatment.

Transmission electron microscopy (TEM) lift-out specimens were prepared in an FEI 3D Quanta dual-beam FIB with a standard lift-out procedure [8]. Analytical (Scanning) TEM (STEM) analysis was carried out in a JEOL ARM 200CF operated at 200 kV and equipped with a HAADF detector and a Centurio EDS detector. STEM HAADF imaging provides mass contrast and is ideal for observing grain boundaries, cracks, and voids and highlighting the heavier metal dopant precipitates in the oxide matrix. EDS was then used for compositional analysis of any precipitates observed. X-rays in the range of 0.2-20 or 40keV were captured in each experiment for rare earth L lines. Acquisition and analysis of EDS maps were carried out with Thermo Fisher Scientific Pathfinder 1.4. The compass principal component analysis tool can separate regions of different compositions and conduct a more meaningful compositional analysis.

**Fig. 3:**
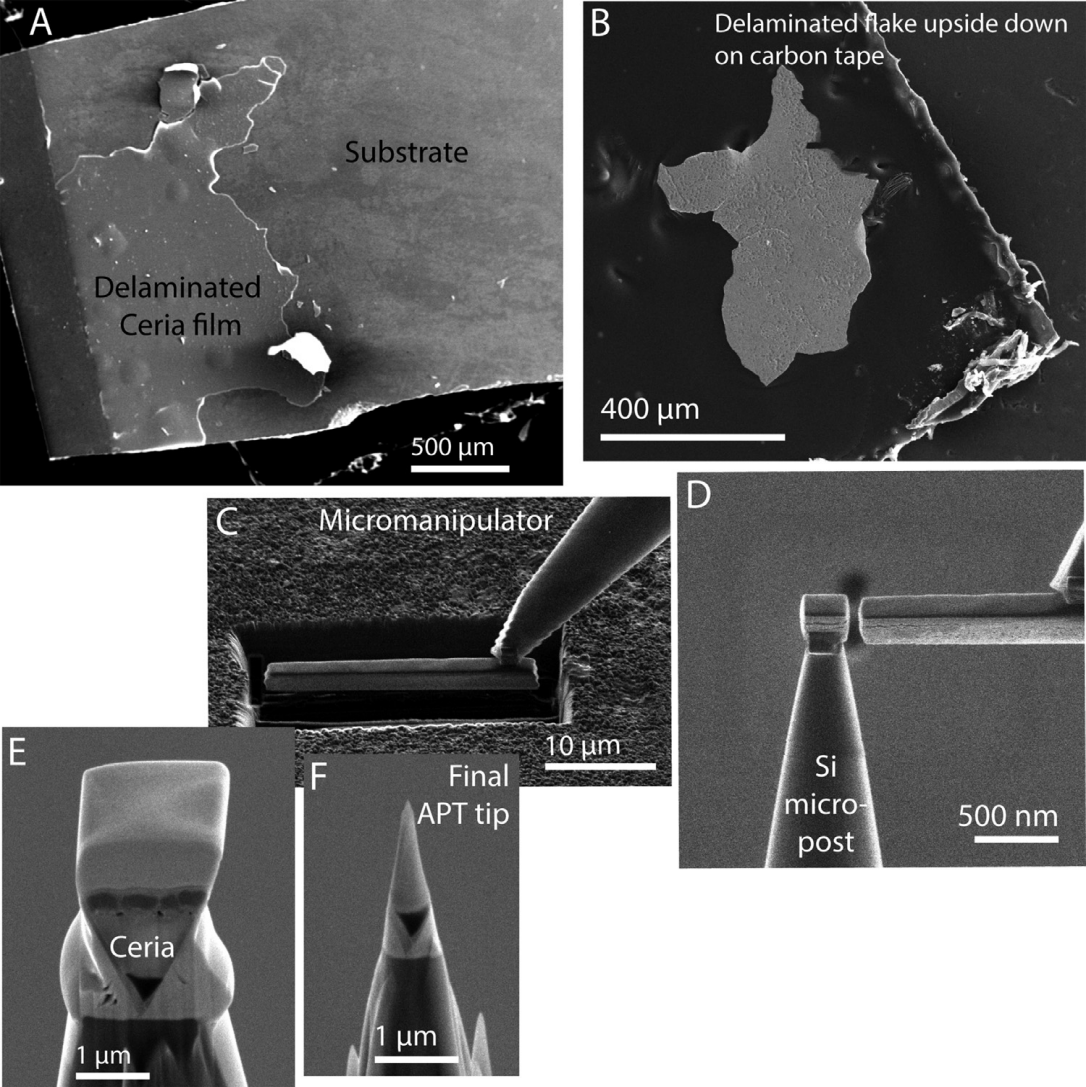
A) SEM image showing the delaminating film of doped ceria after heat treatment at 1100 °C for 10 h. B) flake mounted upside down on carbon tape. C) Material lifted out in the FIB for the atom-probe experiment. D) Attachment to premanufactured Si micropost array. E) Sideview of the sample wedge before sharpening. F) Final needle specimen before the atom-probe experiment.

For Sample 7, the doped ceria film delaminated from the substrate during heat treatment. TEM analysis of a sample extracted from the part of the film still attached to the substrate (see left side of Fig. 3A) revealed many precipitates close to the interface between the film and substrate. Therefore, APT analysis was attempted on this interface region. For this purpose, a peeled-off flake was mounted with the original surface on a piece of carbon tape and the presumably precipitate-covered side up. The sample was sputter-coated with 100 nm of Ti metal using a Cressington 208HR magnetron sputter coater at ∼10^−2^ Torr. The conductive coating prevented charging under the electron beam and provided extra material at the new, rough surface. Needle-shaped APT specimens were prepared using the FEI 3D Quanta dual-beam FIB. Fig. 3 shows the lift-out procedure. The sides of the triangular prism lift-out bar were at ∼1.5 µm smaller than usual to avoid including too much of the C tape into the finished atom-probe needles. Fig. 3E shows the layers in the extracted material from bottom to top: carbon tape, ceria, mixed surface precipitates and sputter-coated Ti, electron beam deposited Pt, and finally, ion beam deposited Pt. The final atom-probe tip in Fig. 3F terminates in the precipitate/Ti layer. Standard Si micropost coupons from Cameca Instruments were used to hold FIB lift-outs. APT examinations were carried out in a CAMECA LEAP 4000XHR at 40 K in laser mode (λ = 355 nm), with a pulse rate of 125 – 200 kHz, a pulse energy of 120pJ. The overall detection efficiency of the detector was 36%. Three-dimensional (3D) data reconstruction and analysis of the precipitates were performed with Cameca's Integrated Visualization and Analysis Software (IVAS).

## CRediT Author Statement

**Karen Kruska:** Methodology, Formal Analysis, Investigation, Data Curation, Writing – original draft, Visualization; **Weilin Jiang:** Methodology, Validation, Writing – review & editing, Supervision, Funding acquisition; **Xuemei Wang:** Methodology, Resources; **Lin Shao:** Methodology, Resources, Supervision; **Brian J. Riley:** Methodology, Resources, Writing – review & editing; **Ram Devanathan:** Conceptualization, Writing – review & editing, Project administration, Funding acquisition.

## Declaration of Competing Interest

The authors declare that they have no known competing financial interests or personal relationships which have, or could be perceived to have, influenced the work reported in this article.
